# Intracellular free flavin and its associated enzymes participate in oxygen and iron metabolism in *Amphibacillus xylanus* lacking a respiratory chain

**DOI:** 10.1002/2211-5463.12425

**Published:** 2018-05-09

**Authors:** Shinya Kimata, Daichi Mochizuki, Junichi Satoh, Ken Kitano, Yu Kanesaki, Kouji Takeda, Akira Abe, Shinji Kawasaki, Youichi Niimura

**Affiliations:** ^1^ Department of Bioscience Tokyo University of Agriculture Japan; ^2^ Graduate School of Biological Science Nara Institute of Science and Technology Ikoma Japan; ^3^ Nodai Genome Research Center Tokyo University of Agriculture Japan; ^4^ Teacher Education Course Tokyo University of Agriculture Japan; ^5^ Department of Ophthalmology Sapporo Medical University Hokkaido Japan

**Keywords:** *Amphibacillus xylanus*, flavin reductase, free flavins (non‐protein‐binding flavins), iron, oxygen

## Abstract

*Amphibacillus xylanus* is a recently identified bacterium which grows well under both aerobic and anaerobic conditions and may prove useful for biomass utilization. *Amphibacillus xylanus,* despite lacking a respiratory chain, consumes oxygen at a similar rate to *Escherichia coli* (130–140 μmol oxygen·min^−1^·g^−1^ dry cells at 37 °C), suggesting that it has an alternative system that uses a large amount of oxygen. *Amphibacillus xylanus* NADH oxidase (Nox) was previously reported to rapidly reduce molecular oxygen content in the presence of exogenously added free flavin. Here, we established a quantitative method for determining the intracellular concentrations of free flavins in *A. xylanus*, involving French pressure and ultrafiltration membranes. The intracellular concentrations of flavin adenine dinucleotide (FAD), flavin mononucleotide (FMN), and riboflavin were estimated to be approximately 8, 3, and 1 μm, respectively. In the presence of FAD, the predominant free flavin species, two flavoproteins Nox (which binds FAD) and NAD(P)H oxidoreductase (Npo, which binds FMN), were identified as central free flavin‐associated enzymes in the oxygen metabolic pathway. Under 8 μm free FAD, the catalytic efficiency (*k*_cat_/*K*_m_) of recombinant Nox and Npo for oxygen increased by approximately fivefold and ninefold, respectively. Nox and Npo levels were increased, and intracellular FAD formation was stimulated following exposure of *A. xylanus* to oxygen. This suggests that these two enzymes and free FAD contribute to effective oxygen detoxification and NAD(P)^+^ regeneration to maintain redox balance during aerobic growth. Furthermore, *A. xylanus* required iron to grow aerobically. We also discuss the contribution of the free flavin‐associated system to the process of iron utilization.

**Abbreviations**
CFE: cell‐free extract
DTPA: diethylenetriaminepentaacetic acid
FAD: flavin adenine dinucleotide
FMN: flavin mononucleotide
HPLC: high‐performance liquid chromatography
IDA: iminodiacetic acid
Nox: NADH oxidase
Npo: NAD(P)H oxidoreductase
NTA: nitrilotriacetic acid
PDF: peptide deformylase
Prx: peroxiredoxin

*Amphibacillus xylanus* was isolated as a biomass utilization bacterium that can assimilate xylan under alkaline conditions in the presence or absence of oxygen 1. This bacterium showed unique phenotypic and chemotaxonomic characteristics; therefore, it was accepted as a new genus (*Amphibacillus*) and species (*xylanus*) 2, 3. Presently, nine species belong to this genus 2, 4, 5, 6, 7, 8, 9, 10. *Amphibacillus xylanus* grows well under aerobic and anaerobic conditions, exhibiting the same growth rate and cell yields under both conditions, despite its lack of a respiratory chain 2. The aerobic and anaerobic metabolic pathways in this species produce the same amount of adenosine triphosphate (ATP) under their respective growth conditions 11, 12.

A flavoprotein, NADH oxidase (Nox), was purified as an oxygen metabolic enzyme that reduces oxygen to form hydrogen peroxide (H_2_O_2_) in the aerobic metabolic pathway of *A. xylanus* 13. In addition, Nox can transfer a hydride ion from NADH to the active site of AhpC, currently known as peroxiredoxin (Prx) 14, and belongs to a Prx reductase family that includes AhpF 15. The Prx enzyme is a cysteine‐based peroxidase and contains a redox‐active disulfide that catalyzes the reduction in hydroperoxides. Thus, *A. xylanus* Nox can reduce H_2_O_2_ to water in the presence of Prx at the expense of additional NADH. The *K*_m_ value of Nox for Prx was revealed to be 8.9 μm 16, while that for oxygen was 1.7 mm, which is too high to effectively catalyze reoxidation of NADH by oxygen in cells 17. In the presence of additional flavin adenine dinucleotide (FAD), however, NADH oxidase activity was markedly accelerated and the apparent *K*_m_ value for oxygen markedly decreased 16. This is likely because Nox oxidizes NADH to NAD^+^ at a high rate by efficiently reducing FAD as a substrate rather than oxygen. Moreover, it is known that reduced flavin is easily oxidized by molecular oxygen 18, 19. Therefore, flavin reductases can transfer reducing equivalents of NAD(P)H to oxygen efficiently via reduced flavin. These observations emphasize the importance of intracellular flavin (free flavin) and flavin reductase in *A. xylanus*. Our study focused on the presence and role of free flavin in the metabolic pathway of *A. xylanus*.

Flavin adenine dinucleotide, flavin mononucleotide (FMN), and riboflavin are the general flavin species present in cells. FAD and FMN bind to flavoproteins and function as their cofactors. Several biological reactions that require free FAD or FMN have been reported in bacterial cells 4, 17, 20, 21, 22, 23, 24, 25, 26, 27, 28, 29, 30, 31, 32, 33, 34, 35, 36. Intracellular free flavins have been evaluated in *Escherichia coli* 37. However, their existence in the cells of bacteria has not been validated because of the lack of effective methods for distinguishing between flavins in their free form and protein‐binding form. We previously separated free flavin from protein‐binding flavin in *A. xylanus* using a gel‐filtration column 17. However, this method was inadequate for quantification, as free flavins nonspecifically interacted with the column carriers, leading to variations in the yield of free flavins. Alternatively, an ultrafiltration membrane was used to prepare protein‐free fractions 38, 39.

In this study, we established a quantitative method for determining the concentrations of free flavins in *A. xylanus* involving an ultrafiltration membrane. Based on the physiological concentration of free flavin, the free FAD‐associated oxygen metabolic enzyme system was identified in *A. xylanus*. Additionally, this bacterium required both iron and oxygen to grow under aerobic conditions. This study suggests the physiological significance of free flavin and its associated enzymes in oxygen and iron metabolism in *A. xylanus*.

## Materials and methods

### Bacterial strains and growth

*Amphibacillus xylanus* Ep01 was grown in alkaline glucose/yeast (GY) medium 1 at 39.5 °C. Normal aerobic cultivation was performed in a glass flask containing 1 or 1.5 L GY medium with circular agitation at 150 rpm. The cultures were grown under various atmospheric concentrations of oxygen at 0, 10, 21, or 40% in a jar fermenter (MDL‐1001S, B.E. Marubishi, Pathumthani, Thailand) containing 3 L GY medium, supplied with the corresponding oxygen (O_2_)/nitrogen (N_2_)‐mixed gases (0%: 100% N_2_; 10%: 10% O_2_/90% N_2_; 21%; 21% O_2_/79% N_2_; 40%: 40% O_2_/60% N_2_) at 0.5 volume·volume^−1^·min^−1^ and agitated at 100 rpm. For iron‐limited cultures, the glass flasks were washed with 0.1 m nitric acid before culturing to prevent iron contamination. Alkaline semidefined medium (pH 9.7) was used rather than GY medium to control iron content. The regular composition containing 1 ppm iron was as follows (L^−1^): 2 g ammonium nitrate, 1 g K_2_HPO_4_, 3.75 g casamino acid, 20 mg tryptophan, 0.2 mg biotin (+), 0.2 mg folic acid, 1 mg pyridoxine hydrochloride, 0.5 mg riboflavin, 0.5 mg thiamine hydrochloride, 0.5 mg nicotinic acid, 0.5 mg calcium (+)‐pantothenate, 0.1 mg cyanocobalamin, 0.5 mg *p*‐aminobenzoic acid, 0.25 mg orotic acid monohydrate, 0.25 mg thymidine, 0.25 mg inosine, 0.1 mg (±)‐thioctic acid, 250 mg L(+)‐ascorbic acid sodium salt, 10 mg adenine, 10 mg guanine hydrochloride, 10 mg uracil, 10 mg xanthine, 50 mg L(−)‐cysteine, 0.2 g MgSO_4_·7H_2_O, 5 mg MnSO_4_·5H_2_O, 5 mg FeSO_4_·7H_2_O, 5 mg ZnSO_4_·7H_2_O, 0.1 g CaCl_2_·2H_2_O, 0.5 mg CuSO_4_·5H_2_O, 1 mg resazurin sodium salt, 120 mL 25% D(+)‐glucose, 100 mL 1 m NaHCO_3_‐NaH_2_CO_3_ (pH 10.6), and 780 mL ultrapure water. For iron‐free conditions, FeSO_4_·7H_2_O was absent from the medium. The semidefined medium was also used for experiments investigating H_2_O_2_ production by *A. xylanus*; however, L(+)‐ascorbic acid sodium salt and resazurin sodium salt were absent and 2 g ammonium nitrate was replaced with 5.7 g diammonium hydrogen citrate, as these reagents sometimes interfere with assays for H_2_O_2_. *Escherichia coli* JM109 and *Lactobacillus plantarum* WCFS1 were grown at 37 °C in LB broth and GYP medium in glass flasks, respectively, with circular agitation at 150 rpm. *Escherichia coli* DE3 or *E. coli* JM109 harboring expression plasmids were aerobically cultured in LB broth supplemented with 50 μg·mL^−1^ ampicillin at 37 °C.

### Oxygen consumption rate of resting cells from bacteria

*Amphibacillus xylanus* Ep01, *E. coli* JM109, and *L. plantarum* WCFS1 were aerobically grown in 1 L of corresponding medium as described above. To obtain fresh cells, cells were harvested as soon as possible at 25 °C when optical density (OD)_660_ equaled 1.0, and were subsequently suspended with 20 mL of 50 mm sodium carbonate, pH 9.6 for *A. xylanus* or 50 mm sodium phosphate, pH 7.0 for *E. coli* and *L. plantarum* (50‐fold concentrated). The suspensions were gently agitated at room temperature to maintain aerobic conditions until analysis. Oxygen consumption by each resting cell type was evaluated using a Clark‐type oxygen electrode (model 5331; Yellow Springs Instruments Co., Yellow Spring, OH, USA) between 30 and 45 °C. Prior to the assays, buffers were bubbled with air for 15 min at each temperature. The reaction was initiated by adding 40 μL of cell suspension in 1960 μL of air‐saturated buffer supplemented with 1% glucose (corresponding to OD_660_ = 1.0). Activities were determined as the amount of dissolved oxygen consumed by 1 g of dry cells per 1 min.

### Cell homogenate methods for extraction of intracellular free flavins in *Amphibacillus xylanus*

The aerobically cultured cell pellet was suspended in 4 volumes of 50 mm sodium phosphate (pH 7.0) and used as the sample. This sample was homogenized using either an ultrasonic cell disruptor (Sonifier 450D from Branson, Danbury, CT, USA) with conditions of a 0.1 pulse and 50% duty at 4 °C for 2.5–20 min or by passing through a French pressure cell (FA078A from SLM‐AMINCO; Thermo Fisher Scientific, Inc., Waltham, MA, USA) 1–4 times at 140 MPa with cooling on ice after each passage. The cell‐free extracts (CFEs) were obtained after the removal of cell debris from these samples by centrifugation at 12 000 × *g* and 4 °C for 20 min. The protein concentration in CFEs was determined by the Bradford method. NADH oxidase activity in CFEs was determined in 50 mm sodium phosphate, including 100 μm NADH, at 25 °C (pH 7.0). The free FAD content in CFEs was evaluated as described below.

### Quantification of intracellular free flavins in *Amphibacillus xylanus*

The cells were harvested at 12 000 ***g*** and 4 °C when the OD_660_ reached approximately 1.0, and then washed three times with 50 mm sodium phosphate (pH 7.0). The cell pellet was resuspended in 4 volumes of the above buffer, and the suspension was divided into two groups: one was used for the extraction of free flavins, while the other one was used for the determination of wet cell weight and dry cell weight in the cell suspension. Because these weight values were needed to estimate the intracellular concentration of flavins, the sample volume was recorded before and after the centrifugation step described in the following extraction procedures.

An aliquot of sample was passed through the French pressure cell 3 times at 140 MPa. The extract was centrifuged at 12 000 ***g*** and 4 °C for 20 min to obtain the CFE. The CFE was centrifuged at 100 000 ***g*** and 4 °C for 2 h. The supernatant was passed through a 30‐kDa MWCO membrane (Nanosep^®^ Centrifugal Devices; Pall Corporation, Port Washington, NY, USA) at 14 000 × *g* and 4 °C, and then, the flow‐through was passed through a 3‐kDa MWCO membrane (Nanosep^®^ Centrifugal Devices; Pall Corporation) at 14 000 ***g*** and 4 °C. In this study, the flow‐through fraction was defined as the free flavin fraction. In contrast, the CFE was considered as the total flavin fraction that included the free form and protein‐binding form. Next, 100 μL of the total or free flavin fraction was thoroughly mixed with 400 μL methanol and incubated at 60 °C for 23 min. The supernatant was collected after centrifugation at 20 400 ***g*** and 4 °C for 15 min, and dried under N_2_ gas at room temperature. The dried sample was dissolved in 50 μL of 5 mm ammonium acetate.

These samples (20 μL) were applied to an HPLC system (pump L‐7100; Hitachi, Tokyo, Japan, UV‐VIS detector; Hitachi, FL spectrophotometer S‐3370; Soma, Tokyo, Japan). Ammonium acetate (5 mm)/methanol buffer mixed at a ratio of 7 : 3 was used as the mobile phase. The flavins in the sample were separated using a PEGASIL ODS column (4.6 ø × 250 mm; Sensyu Scientific, Tokyo, Japan) at 50 °C and a flow rate of 1 mL·min^−1^ and were detected at excitation and emission wavelengths of 450 and 530 nm, respectively. Standard mixtures containing FAD, FMN, and riboflavin at respective concentrations of 0.2–10, 0.1–5, and 0.04–2 μm were also analyzed to obtain calibration curves. Data sets of HPLC were analyzed by chromato‐pro version 3.0.0 (Runtime, National Instruments Corporation, Austin, TX, USA). The flavin contents in *A. xylanus* were expressed as nmol of flavin per 1 g of dry cell, and the intracellular concentration was estimated from the weight ratio of the wet and dry cells (g wet cell–g dry cell·g^−1^ dry cell = mL water·g^−1^ dry cell).

Wet cell weight in the sample was determined as follows: an aliquot of the cell suspension obtained as described above was centrifuged at 48 380 × *g* and 4 °C for 20 min, and the cell pellet was weighed after removing the supernatant. This procedure was repeated until the weight decreased linearly with centrifugations, and a linear function (*y* = *ax* + *b*) between weights (*y*‐axis) and the number of measurements (*x*‐axis) was obtained. The *y*‐intercept (*b*) value was defined as the wet cell weight in the cell suspension. Dry cell weight was determined by drying the wet cells at 105 °C until the weight no longer changed.

### Chromatography of free flavin‐associated oxygen metabolic enzymes from *Amphibacillus xylanus*

NAD(P)H oxidases activated by 10 μm FAD were fractionated from the soluble fraction of *A. xylanus* by column chromatography. The soluble fraction was prepared from approximately 50 g of cells as described previously 13, except that ammonium sulfate precipitation was performed at a concentration of 200 g·L^−1^. Enzyme fractionation was performed using the same columns and corresponding elution conditions as described previously 40. Briefly, the active fraction obtained after passing through a butyl‐Toyopearl 650S column (3.5 × 45 cm, Tosoh, Tokyo, Japan) was applied to a DEAE Sepharose fast flow column (3.5 × 23 cm, GE Healthcare, Little Chalfont, UK), and then, enzymes were eluted using a NaCl gradient from 0 to 300 mm. The NAD(P)H oxidase activities of these samples were determined in 50 mm sodium phosphate containing 100 μm NAD(P)H in the presence or absence of 10 μm FAD (pH 7.0) at 25 °C.

### Overexpression and purification of NADH oxidase and NAD(P)H oxidoreductase

Recombinant Nox from *A. xylanus* was overexpressed and purified as described previously 17.

The overexpression system for Npo in *E. coli* DE3 was constructed using the Champion™ pET101 Directional TOPO^®^ expression kit (Invitrogen; Thermo Fisher Scientific, Inc., Waltham, MA, USA). The gene for Npo which was to be cloned into the pET101 vector was amplified by polymerase chain reaction (PCR) using chromosomal DNA from *A. xylanus* as a template, PrimeStar HS DNA polymerase (Takara, Shiga, Japan), and the following primer pairs: 5′‐CACCATGGCAAAAGATTTT‐3′ (forward) and 5′‐TTATTTAAAATTTTCACAC‐3′ (reverse). The overexpression of Npo protein in *E. coli* DE3 was induced by adding 0.5 mm IPTG when the OD_660_ reached approximately 0.6. Following an additional 3 h of cultivation, the cells were harvested by centrifugation and stored at −80 °C until use. Recombinant Npo was purified as described above. The extinction coefficient was determined to be ε441 = 13 800 m ^−1^·cm^−1^ by spectral analysis of free FMN, which was released from Npo protein following addition of 0.1% SDS 41.

### Kinetics parameters for FAD and oxygen

The kinetics parameters for FAD were evaluated in 50 mm sodium phosphate buffer (pH 7.0) containing 0.5 mm EDTA, 150 μm NAD(P)H, and various concentrations of FAD (15–150 μm) at 25 °C. The reaction was initiated by adding Nox or Npo enzymes, and the activity was calculated as the decrease in absorbance at 450 nm corresponding to reduced FAD formation. NADH and NADPH were used as electron donors for Nox and Npo, respectively. The kinetic parameters for oxygen in the presence or absence of 8 μm free FAD were also determined in the same standard reaction mixture containing varying concentrations of oxygen (125–1252 μm) at 25 °C. Activity was calculated as the decrease in an absorbance at 340 nm corresponding to NAD(P)H oxidation. The *K*_m_ and *k*_cat_ values were estimated by nonlinear regression analysis of the initial reaction velocity versus substrate concentration using the Michaelis–Menten equation using sigmaplot version 11 (Systat Software, San Jose, CA, USA).

### Hydrogen peroxide production

Hydrogen peroxide production during catalysis by the Nox‐Prx system and Npo in the presence or absence of 8 μm free FAD was monitored by the addition of catalase using a Clark‐type oxygen electrode as described previously 11, 13. The reactions were performed in air‐saturated 50 mm sodium phosphate buffer containing 0.5 mm EDTA, 300 mm ammonium sulfate, and 150 μm NAD(P)H, with or without 8 μm FAD (pH 7.0). The final concentrations of Nox, Prx, and Npo in the reaction mixtures were 0.1, 30, and 5 μm, respectively. The protein concentration of Nox and Npo was determined using each extinction coefficient of ε_450_ = 13 200 m ^−1^·cm^−1^ for Nox 17 and ε_441_ = 13 800 m ^−1^·cm^−1^. Each extinction coefficient was determined based on the bound flavin of flavoprotein. Nox and Npo bind 1 mol FAD per subunit and 1 mol FMN per subunit, respectively. Recombinant Prx enzyme was purified from *E. coli* as described previously 16, and the protein concentration was determined using an extinction coefficient of ε_279_ = 25 800 m ^−1^·cm^−1^.

*Amphibacillus xylanus* was aerobically grown in semidefined medium. The cells were harvested at room temperature and washed with medium adjusted to pH 8.0. The oxygen consumption of living cells was monitored in a total 2 mL of semidefined medium (pH 8.0) supplemented with 2.5% glucose and *A. xylanus*. When the oxygen consumption was saturated, H_2_O_2_ production in the reaction mixture was determined as described above.

### Ferric reductase activity

The ferric reductase activities of CFE, Nox, or Npo were evaluated aerobically as described before 32. The reaction was performed in 50 mm sodium phosphate buffer containing 150 μm NAD(P)H and 100 μm iron compound, with or without 8 μm FAD (pH 7.0), at 25 °C. The concentration of iron in the five iron compounds used as the substrate was determined by the bathophenanthroline assay.

### Western blotting

Cells of *A. xylanus* grown under various concentrations of oxygen (0, 10, 21, 40%) were passed through the French press at 140 MPa three times. The concentration of total protein in the CFEs was determined using the Bradford method. Next, 2 μg of extracts was transferred to polyvinylidene fluoride membranes (Bio‐Rad, Hercules, CA, USA) after 15% SDS/PAGE. The antigen–antibody reaction and chemiluminescence were performed as described previously 13. Image analysis was performed using the ChemiDoc XRS Plus (Bio‐Rad).

### Northern blotting

*Amphibacillus xylanus* was grown in GY medium under aerobic and anaerobic conditions. The respective cells were harvested when the OD_660_ reached 0.5, and total RNA in the cells was isolated using TRIzol reagent 40. A series of northern blot analyses were performed as described previously 40. The DNA probe for peptide deformylase was amplified by PCR using the following primer pair: 5′‐TTGAAGGGCACCCTGTTCTT‐3′ and 5′‐CCACGCAAGCGTAGTTTGAC‐3′.

## Results and Discussion

### Oxygen consumption activity of *Amphibacillus xylanus*

**Figure 1:**
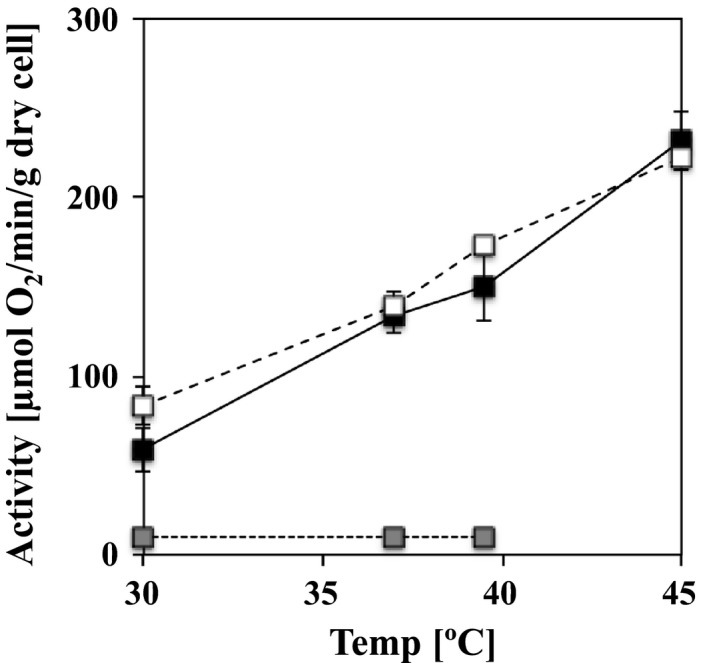
Oxygen consumption rates of bacterial resting cells. The oxygen consumption rate in resting cells *Amphibacillus xylanus* Ep01 (black square), *Escherichia coli* JM109 (white square), and *L. plantarum* WCFS1 (gray square), grown under aerobic conditions, was determined using an oxygen electrode. The assays were performed in 50 mm sodium carbonate (pH 9.7) for *A. xylanus* or 50 mm sodium phosphate for the other two bacterial species in the presence of 1% glucose between 30 and 45 °C. The rates were determined as the amount of dissolved oxygen consumed by 1 g dry cells per 1 min. The data are the mean values of three independent experiments, and error bars indicate standard deviation.

*Amphibacillus xylanus* consumes dissolved oxygen in medium using xylan as a growth substrate during static culture, despite its lack of a respiratory chain 1. This oxygen consumption was also observed in experiments using resting cells cultured aerobically in the presence of glucose as a growth substrate 12. To estimate the oxygen consumption capacity of *A. xylanus*, aerobic resting cells of *A. xylanus*,* E. coli* JM109 with a respiratory chain, and *L. plantarum* WCFS1 lacking a respiratory chain were prepared, and their respective oxygen consumption activities under 1% glucose were compared at various temperatures. *Escherichia coli* JM109 showed markedly higher oxygen consumption activity at temperatures of 30–45 °C compared to *L. plantarum*. WCFS1 (Fig. 1). This high activity is associated with the presence of the respiratory chain. However, *A. xylanus* which lacks this system exhibited nearly the same oxygen consumption activity as *E. coli* JM109 at each temperature analyzed (Fig. 1), indicating that *A. xylanus* has an alternative enzyme system that uses a large amount of oxygen.

This bacterium contains a 55‐kDa flavoprotein, NADH oxidase (Nox), as its major oxygen metabolic enzyme 13. However, its affinity for oxygen is too low considering that the enzyme is purified from the major active fraction in *A. xylanus* cells, as the *K*m value for oxygen of recombinant Nox (1.7 mm) is too high compared to the dissolved oxygen concentration (approximately 260 μm) under standard atmosphere conditions at 25 °C 17. Thus, Nox alone is likely not responsible for this strong oxygen consumption as shown in Fig. 1. In contrast, its apparent affinity for oxygen and oxygen consumption activity are dramatically increased by the addition of free flavin 16, 17. Thus, we hypothesized that an oxygen metabolic system is involved in free flavin utilization in *A. xylanus*, whereas free flavin‐dependent activation is observed under the nonphysiological concentration of 150 μm free FAD 16. In this study, we established a method for quantifying intracellular free flavin.

### Extraction and quantification of intracellular free flavin

Free flavins were fractionated from CFEs using centrifugal membranes that exclude proteins larger than 3 kDa. The yield of filtrates obtained from the standard mixture containing 10 μm of FAD, FMN, and riboflavin showed recoveries of 96 ± 3, 99 ± 1, and 87 ± 3%, respectively. Because at least 78% of flavins were recovered using 1 μm of the standards, we investigated the cell disruption methods used to extract free flavins based on this filtration method.

**Figure 2:**
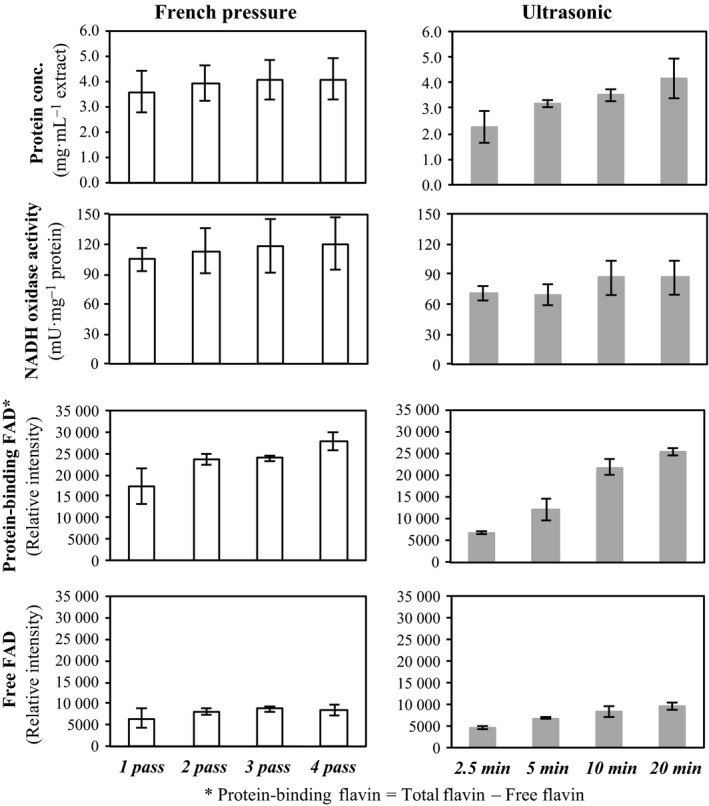
Comparison of extraction methods for intracellular free flavins. The cell‐free extract (CFE) was prepared from *Amphibacillus xylanus* cells using the French pressure cell or an ultrasonic homogenizer at various processing timepoints. The free and protein‐binding FAD contents, total protein concentration, and NADH oxidase activity were monitored in each case. Free flavin, which is a protein‐free fraction, was fractionated from total flavin fractions in CFE by centrifugal membranes and analyzed by HPLC. In this figure, FAD was measured as a representative of flavin. The protein‐binding FAD content was expressed as a theoretical value obtained from experimental values of total and free FAD. Data are presented as the mean values of three independent experiments, and error bars indicate standard deviation.

To disrupt bacterial cells, beads beaters, ultrasonic disruption, and French press are commonly used. Among these methods, beads beaters have the advantage of being able to treat a small amount of sample efficiently in a short time. However, an artificial increase in free flavin content relative to that of the total was observed under the conditions analyzed in the present study (data not shown). Therefore, we prepared CFEs from *A. xylanus* using an ultrasonic homogenizer or French pressure cell at various processing timepoints and monitored the free and protein‐binding FAD contents in each sample. As presented in Fig. 2, the extraction yield of free and protein‐binding FAD reached a saturation plateau within 10–20 min of ultrasonication or 2–4 times of French pressure treatment. Under the same conditions, the total protein extract and NADH oxidase activity were nearly at the saturation level (Fig. 2). These results suggest that ultrasonic and French pressure treatments under their specific conditions were able to extract free flavins efficiently from bacterial cells without artificial removal of the cofactor of flavoproteins. According to the NADH oxidase activity level (Fig. 2), the French press was a more moderate method for the samples compared to the ultrasonic disruptor. In this study, we established that French pressure treatment (performed three times) prior to membrane filtration was the most suitable method for extracting free flavins from *A. xylanus*.

Intracellular free flavins in *A. xylanus* cultured under normal aerobic conditions (21% oxygen) were extracted as described above and analyzed by HPLC. The amounts of FAD, FMN, and riboflavin were 31.4 ± 1.2, 10.8 ± 3.4, and 5.2 ± 3.8 nmol·g^−1^ dry cells, respectively (Table 1). The volume of water in the cells was estimated to be approximately 4 mL·g^−1^ dry cells from the weight ratio of wet and dry cells. Based on these values, the concentrations of free FAD, FMN, and riboflavin were approximately 8, 3, and 1 μm, respectively. The free/total ratios of FAD and FMN were approximately 17% and 10%, respectively. Flavin analysis results revealed that FAD is the major free flavin species in *A. xylanus*. This is consistent with a previous report showing that free FAD is more effective for accelerating the oxygen metabolic activity of *A. xylanus* Nox compared to FMN or riboflavin 17.

**Table 1:** Intracellular flavin contents in *Amphibacillus xylanus**.*** Data are presented as the mean ± standard deviation from three independent experiments and expressed as the nmol flavin per gram of dry cell. The binding flavin contents are theoretical values from the difference between the experimental values of total and free flavin. ND, not detected

Flavin species and the contents	Forms	Oxygen conc.			
		0%	10%	21%	40%
FAD (nmol·g^−1^ dry cell)	Total	70.6 ± 10.9	130.7 ± 32.5	186.9 ± 55.3	193.5 ± 13.2
	Free	16.7 ± 3.4	33.6 ± 1.9	31.4 ± 1.2	40.2 ± 6.6
	Protein‐binding^a^	53.9 ± 8.0	97.1 ± 33.9	155.5 ± 54.3	153.3 ± 13.1
FMN (nmol·g^−1^ dry cell)	Total	50.9 ± 4.9	94.1 ± 26.5	105.0 ± 13.3	110.6 ± 12.1
	Free	4.2 ± 1.1	11.4 ± 4.2	10.8 ± 3.4	9.0 ± 3.6
	Protein‐binding^a^	46.7 ± 4.7	82.7 ± 22.3	94.2 ± 14.4	101.6 ± 13.7
Riboflavin (nmol·g^−1^ dry cell)	Total	ND	9.2 ± 2.5	14.2 ± 10.4	8.4 ± 1.8
	Free	ND	ND	5.2 ± 3.8	2.6 ± 1.8
	Protein‐binding^a^	ND	9.2 ± 2.6	9.0 ± 6.6	5.8 ± 0.8

a Protein‐binding flavin = Total flavin‐Free flavin.

### Oxygen metabolic enzymes stimulated by free FAD

**Figure 3:**
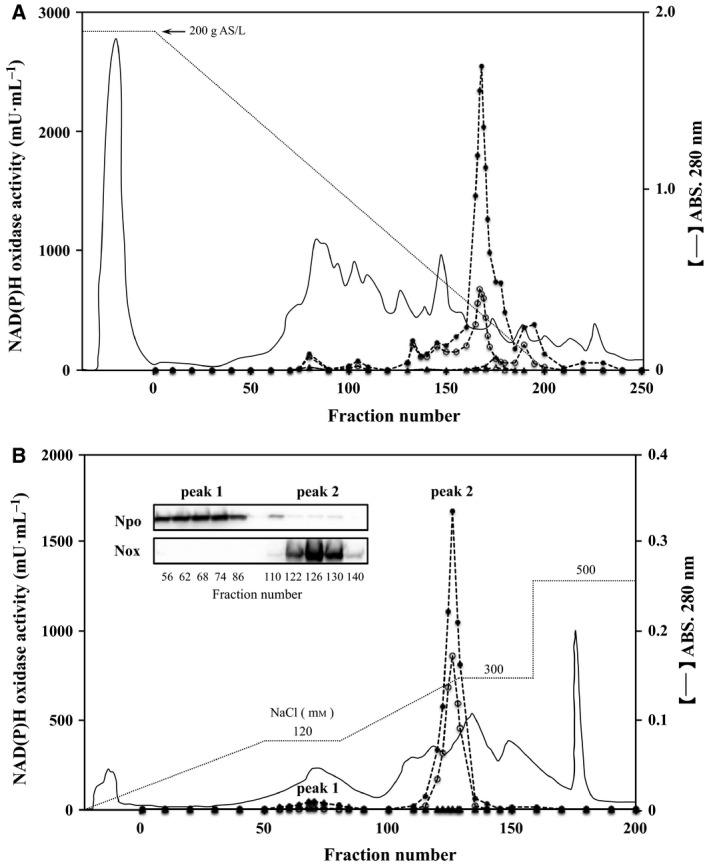
Chromatography of NAD(P)H oxidases stimulated by free FAD. (A) NAD(P)H oxidases in the soluble fraction from *Amphibacillus xylanus* were separated by a butyl‐Toyopearl 650S column with a decreasing concentration of ammonium sulfate (AS) from 200 to 0 g·L^−1^ (dotted line). (B) The active fraction obtained from a butyl‐Toyopearl chromatography (fraction numbers 150–185) was separated by a DEAE Sepharose fast flow column with NaCl gradients from 0 to 120 mm, and subsequently from 120 to 300 mm. Peaks 1 and 2 are fractions containing NAD(P)H oxidoreductase (Npo) and NADH oxidase (Nox), respectively, according to results of western blot analysis using their specific antibodies as presented in the inset. NAD(P)H oxidase activities in each fraction were evaluated in 50 mm sodium phosphate containing 100 μm NAD(P)H with or without 10 μm FAD (pH 7.0) at 25 °C. (○), NADH oxidase activity; (●), NADH oxidase activity with 10 μm FAD; (▵), NADPH oxidase activity; (▲), NADPH oxidase activity with 10 μm FAD.

Previous genomic analysis of *A. xylanus* revealed that this bacterium has two genes for H_2_O_2_‐forming (*nox1*) and H_2_O‐forming (*nox2*) NADH oxidases (Noxs), as well as one gene for NAD(P)H oxidoreductase (*npo*) 40, whereas the gene named *fre* encoding flavin reductase is not found in its genomic sequence. However, oxygen metabolic enzymes stimulated by free flavin should exhibit NAD(P)H‐dependent flavin reductase activity. To identify the free flavin‐associated oxygen metabolic enzymes, namely NAD(P)H‐dependent oxygen/flavin reductase in *A. xylanus*, we investigated NAD(P)H oxidases activities, which were stimulated by 10 μm FAD in the soluble fraction. A major active peak showing oxidase activities for NADH and NADPH was observed following butyl‐Toyopearl 650S column chromatography (fraction numbers 150–185 in Fig. 3A). This peak accounted for approximately 80% of NADH oxidase activity and 50% of NADPH oxidase activity of the whole soluble fraction (Table 2), and no other prominent peaks were observed. When applied to a DEAE Sepharose fast flow column, the major active fraction was divided into two active peaks, and both oxidase activities were promoted by the addition of 10 μm free FAD (Fig. 3B). One peak (peak 1) demonstrated similar activities for NADH and NADPH as electron donors, while the other (peak 2) showed activity only for NADH which was approximately 13‐fold higher compared than that of peak 1 (Table 2). This chromatography pattern is consistent with the results of a previous study of the purification of oxygen metabolic enzymes from *A. xylanus* without free flavin, where NAD(P)H oxidoreductase (Npo) and H_2_O_2_‐forming Nox were purified from peak 1 and peak 2, respectively, while H_2_O‐forming Nox was not found, likely because of its very low activity 40. Based on these observations, we performed western blot analysis using specific antibodies against the Npo and Nox proteins. As expected, the band intensity of each protein was increased with its activity (Fig. 3B). This result suggests that the predominant free flavin‐related oxygen metabolic enzyme in peak 1 was Npo, while that in peak 2 was Nox. Npo is a 23‐kDa flavoprotein (Fig. S1) that binds 1 mol FMN per subunit. Nox is an FAD‐binding protein that was characterized in a previous study 13.

**Table 2:** Chromatography investigation of major oxygen metabolic enzymes stimulated by free FAD in *Amphibacillus xylanus*. Activities were assayed in 50 mm sodium phosphate, including 100 μm NADH or NADPH (pH 7.0), at 25 °C, and were calculated as the decrease in absorbance at 340 nm corresponding to the NAD(P)H oxidation reaction. Total activity (U) represents μmol NAD(P)H·min^−1^. ND = not detected

Step	NADH				NADPH			
	No addition		10 μm FAD		No addition		10 μm FAD	
	Total activity (U)	Yield (%)	Total activity (U)	Yield (%)	Total activity (U)	Yield (%)	Total activity (U)	Yield (%)
Soluble fraction	124.4	100	165.2	100	14.9	100	18.2	100
Butyl‐TOYOPEARL 650S	65.9	53.0	135.1	81.8	4.9	32.9	9.9	54.4
DEAE Sepharose fast flow								
1st peak: NAD(P)H oxidoreductase	1.5	1.2	7.1	4.3	1.5	10.1	7.2	39.6
2nd peak: NADH oxidase	54.2	43.6	92.3	55.9	ND	0	ND	0

### Enzymatic properties of free FAD‐associated oxygen metabolic enzymes

**Figure 4:**
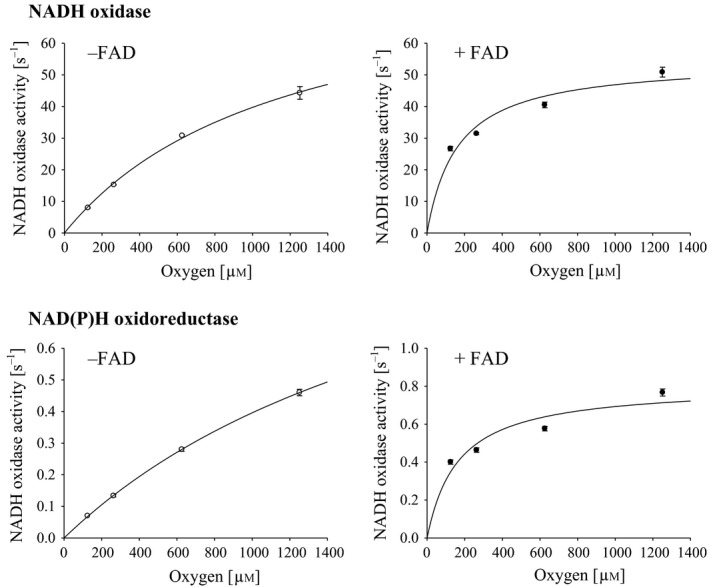
Effect of free FAD at a physiological concentration on NAD(P)H oxidase activities of oxygen metabolic enzymes under various oxygen concentrations. Turnover studies of NADH oxidase or NAD(P)H oxidoreductase were performed in 50 mm sodium phosphate containing 0.5 mm EDTA and 150 μm NADH for NADH oxidase or NADPH for NAD(P)H oxidoreductase in the presence (●) or absence (○) of 8 μm FAD (pH 7.0), with varying dissolved oxygen concentrations from 125 to 1252 μm at 25 °C. Activity was determined by evaluating the decrease in absorbance at 340 nm corresponding to NAD(P)H oxidation and expressed as μmol NAD(P)H oxidation·μmol^−1^ enzyme/s (s^−1^). Data are the mean values of three independent experiments, and error bars indicate standard deviation.

**Figure 5:**
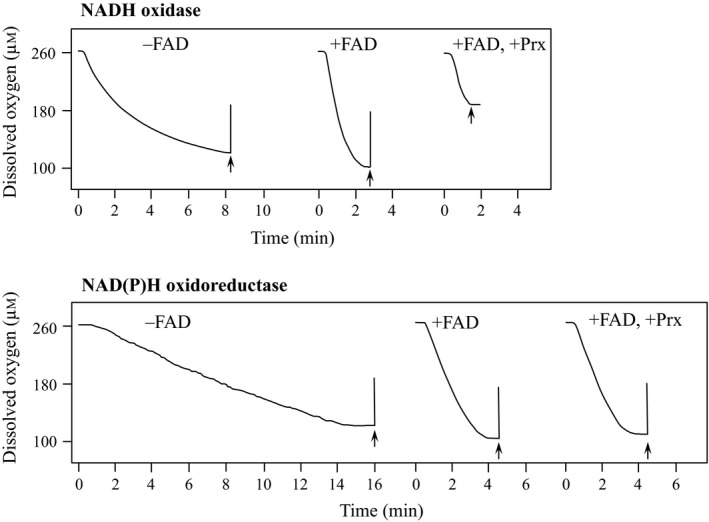
Hydrogen peroxide elimination by NADH oxidase and NAD(P)H oxidoreductase in the presence of free FAD and/or peroxiredoxin (Prx). Oxygen consumption by NADH oxidase or NAD(P)H oxidoreductase was monitored in 50 mm sodium phosphate supplemented with 0.5 mm EDTA, 300 mm ammonium sulfate, and 150 μm NADH for NADH oxidase or 150 μm NADPH for NAD(P)H oxidoreductase with or without 8 μm FAD (pH 7.0). The reactions in the presence of both 8 μm FAD and 30 μm Prx were also performed under the same buffer conditions. At timepoints following the saturation of oxygen consumption, 60 μg of catalase was added to the reaction mixtures, as indicated by the arrowhead. If hydrogen peroxide (H_2_O_2_) was present in the reaction mixture, approximately half an equivalent of oxygen (O_2_) regeneration was observed by addition of catalase according to the following reaction; H_2_O_2_→1/2 O_2_ + 1/2 H_2_O.

The NAD(P)H oxidase activities of recombinant Nox and Npo were evaluated under 21% oxygen in the presence or absence of free FAD at a physiological concentration (8 μm) and 25 °C. All assays were performed with FAD as an additional flavin source because Nox and Npo preferred FAD to FMN for the enhancement of their activities at respective physiological concentrations of 8 and 3 μm (data not shown). The addition of free FAD increased the NADH oxidase activity of Nox by twofold (15.2, 31.4 s^−1^) and NADPH oxidase activity of Npo by 3.5‐fold (0.13, 0.46 s^−1^). These activations were also observed under various concentrations of oxygen from 125 to 1252 μm and were more prominent at lower oxygen concentrations (Fig. 4). The *K*_m_ values for oxygen of Nox and Npo decreased from 1540 ± 210 to 160 ± 10 μm and 2350 ± 520 to 170 ± 30 μm, respectively, with the addition of 8 μm free FAD. This apparent increase in affinity for oxygen led to increased catalytic efficiency *k*_cat_/*K*_m_ of Nox and Npo by approximately five‐ and ninefold, respectively (Table 3). However, the decrease of *K*_m_ for oxygen by free FAD does not mean that free FAD enhanced the oxygen reductase activity of the enzymes themselves, but relate to an enzyme reaction and a secondary chemical reaction that free FAD is reduced by the enzymes and subsequently reduces molecular oxygen. Thus, *K*_m_ and *k*_cat_ estimated from the data of Fig. 4 were not reliable values derived from the enzymes themselves, while activation under 8 μm free FAD conditions was observed for both enzymes by directly monitoring their oxygen consumption using an oxygen electrode (Fig. 5). These results suggest that the physiological concentration of free FAD was effective for accelerating the reduction in molecular oxygen in *A. xylanus*.

**Table 3:** Effect of free FAD at physiological concentrations on kinetics parameters of oxygen on NADH oxidase and NAD(P)H oxidoreductase. Data are presented as the mean ± standard deviation from three independent experiments

Enzyme	Substrate	*K*_m_ (μm)	*k*_cat_ (s^−1^)	*k*_cat_/*K*_m_ (mm ^−1^·s^−1^)
NADH oxidase	O_2_	1540 ± 210	106 ± 11	69 ± 2
	O_2_ + 8 μm FAD	160 ± 10	55 ± 3	335 ± 13
	FAD	37 ± 3	91 ± 8	2423 ± 74
NAD(P)H oxidoreductase	O_2_	2350 ± 520	1.3 ± 0.2	0.6 ± 0.1
	O_2_ + 8 μm FAD	170 ± 30	0.8 ± 0.1	5.1 ± 0.6
	FAD	71 ± 10	1.0 ± 0.1	14 ± 1

Because these accelerations depend on free FAD, its reduced form FADH_2_ should be involved in the oxygen reduction reaction; therefore, we compared the kinetic parameters of FAD and oxygen for Nox and Npo. In our previous experiments, the parameters for NADH and oxygen of Nox were determined by evaluating NADH oxidation with varying electron donors and acceptor concentrations 17. As the *K*_m_ for NADH of Nox and for NADPH of Npo were below 33 μm, the kinetics for FAD were analyzed by fixing the NAD(P)H concentrations at 150 μm and varying the FAD concentration. The *K*_m_ values for FAD of NADH oxidase and NAD(P)H oxidoreductase were estimated to be 37 ± 3 and 71 ± 10 μm, respectively, and these respective *k*_cat_/*K*_m_ values for FAD were several tens of times higher than those for oxygen (Table 3). These results indicate that the reactivity for FAD of the two flavoproteins was sufficiently high compared to that for oxygen. Thus, Nox and Npo function as flavin reductases and were predominant as the major active peaks in Fig. 3A showing flavin reductase activity using 10 μm FAD as the substrate. It is known that reduced flavins show high reactivity for molecular oxygen 4, 36 and therefore rapidly reduce it to H_2_O_2_. As a result, both enzymes reduced molecular oxygen more efficiently via reduced free FAD than by reducing it directly.

**Figure 6:**
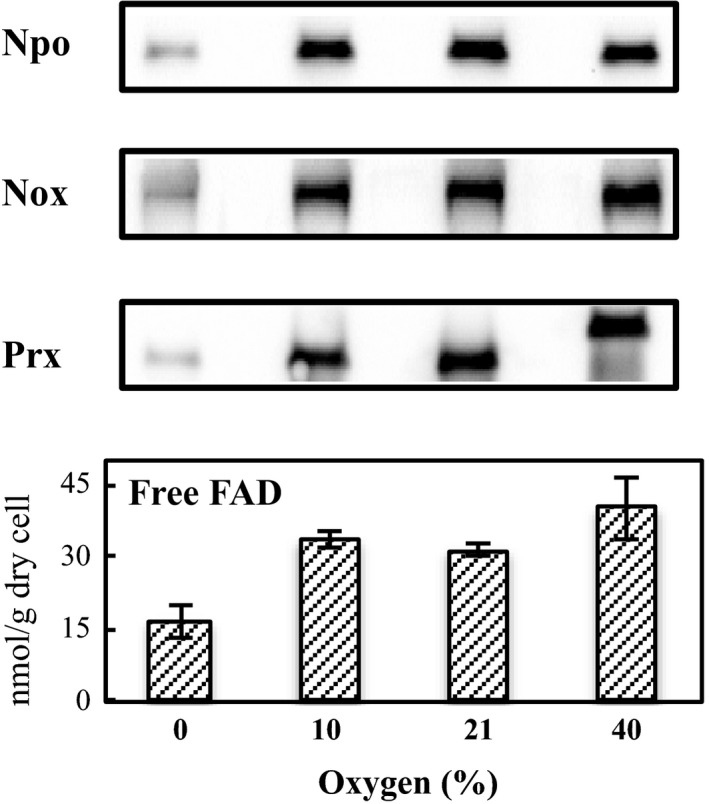
Expression analysis of oxygen metabolic enzymes and free FAD at various oxygen concentrations from 0 to 40%. Western blotting analysis was performed for total protein from *Amphibacillus xylanus* that reacted with anti‐NADH oxidase (Nox) antibody, anti‐peroxiredoxin (Prx) antibody, or anti‐NAD(P)H oxidoreductase (Npo) antibody. The proteins in cell‐free extracts were obtained from *A. xylanus* cells grown under 0, 10, 21, and 40% oxygen levels. Using the same bacterial cells, intracellular free FAD contents were also evaluated.

We previously observed that in *A. xylanus* the genes for Nox, Npo, and numerous flavin synthetic enzymes were induced under aerobic conditions 40. In the present study, the expression levels of Nox and Npo proteins and free flavins under various oxygen concentrations of 0, 10, 21, and 40% were analyzed (Fig. 6). Nox and Npo proteins were robustly expressed under at least 10% oxygen, and their expression levels were maintained between 10 and 40% oxygen. The intracellular free FAD content also increased with an increase in oxygen percentage, showing a similar trend as oxygen metabolic enzymes. These observations suggest that free FAD and the two associated enzymes contribute to effective oxygen consumption during the aerobic growth of *A. xylanus*.

However, Nox and Npo produced H_2_O_2_ during oxygen consumption in the presence of free FAD (Fig. 5). Therefore, H_2_O_2_ production of *A. xylanus* cells in an aerobic resting state was investigated. After the consumption of approximately 200 μm oxygen by the cells, the resultant H_2_O_2_ was too low to be detected by the general method using catalase (data not shown), indicating that the living cells can also reduce oxygen to water. *Amphibacillus xylanus* uses the Nox‐Prx system as an effective H_2_O_2_ scavenger despite its lack of a heme catalase or Mn catalase, NADH peroxidase, and glutathione peroxidase 1, 40.

**Figure 7:**
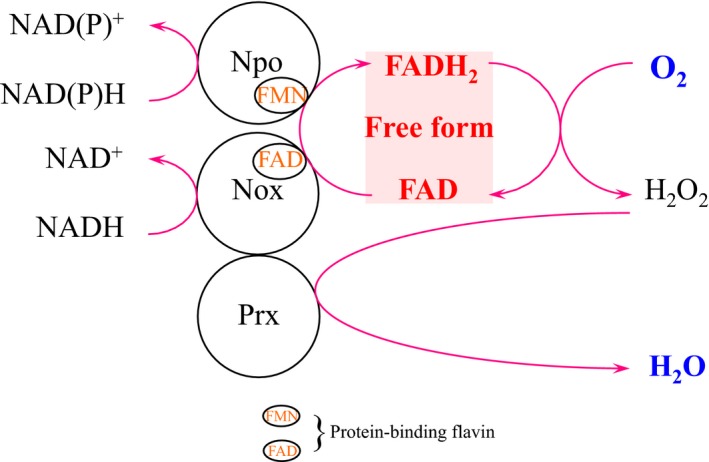
Proposed oxygen metabolic pathway involved in free FAD in *Amphibacillus xylanus*. FAD‐bound NADH oxidase (Nox) and FMN‐bound NAD(P)H oxidoreductase (Npo) reduce free FAD to free FADH_2_ using the substrates NADH or NAD(P)H as electron donors, respectively. Free FADH_2_ efficiently catalyzes the nonenzymatic reduction in molecular oxygen to hydrogen peroxide, and subsequently, oxidized free FAD is regenerated. Hydrogen peroxide is reduced to water by the NADH oxidase‐Prx system.

Because *A. xylanus* Nox is also characterized as a Prx reductase 15, it can catalyze NADH‐dependent H_2_O_2_ reduction to water in cooperation with Prx (Nox‐Prx system) and in the absence of free FAD 15. Prx protein expression was also highly induced by exposure to oxygen along with Nox, Npo, and free FAD (Fig. 6). Thus, we analyzed whether Nox or Npo with Prx could eliminate H_2_O_2_ in the presence of 8 μm free FAD. Npo along with Prx reduced H_2_O_2_ to a low extent, whereas Nox with Prx effectively reduced H_2_O_2_ (Fig. 5). The remaining H_2_O_2_ was too low to be determined by the addition of catalase (Fig. 5), indicating that the Nox‐Prx system efficiently reduced H_2_O_2_ to water in the presence of 8 μm free FAD. Based on these results, the free flavin‐associated oxygen detoxifying system in *A. xylanus* is likely composed of Nox, Prx, Npo, and free FAD, as presented in Fig. 7.

### Aerobic metabolism involved in free FAD in *Amphibacillus xylanus*

*Amphibacillus xylanus* lacks heme synthesis genes, but conserves certain genes that express iron‐associated proteins, two of which are ferredoxin (*fer*) and 7 Fe ferredoxin (*fdxA*) genes. These genes are upregulated under 21% oxygen 40. In this study, we found that *A. xylanus* required iron during aerobic growth (Fig. S2). Correspondingly, the mRNA expression of the iron‐containing enzyme gene encoding peptide deformylase (PDF) was also induced (Fig. S3). PDF is essential for the maturation of polypeptides in bacteria. However, it becomes inactive upon exposure to molecular oxygen due to binding of ferrous ion 42. Thus, continuous expression of PDF holo‐enzyme may be required during aerobic growth of *A. xylanus*.

Iron‐binding proteins, including PDF, always incorporate iron in the form of ferrous ion 28. Therefore, ferric reductase activity is important for activation of these enzymes. The CFE from *A. xylanus* showed NAD(P)H‐dependent ferric reductase activities, which were increased by twofold to threefold in the presence of 8 μm free FAD (Table 4). A free flavin‐dependent increase in ferric reductase activity was also observed in CFEs from other bacteria such as *E. coli* and several organisms, and flavin reductases were purified as contributors to this reaction 28, 32, 33, 34, 35. Nox and Npo, which function as major flavin reductases, showed markedly low ferric reductase activities for ferric citrate and four other synthetic iron compounds. However, their activities toward all substrates were increased after the addition of 8 μm free FAD (Table 4). This prominent increase in ferric iron reductase activity may also be responsible for reduced free FAD (FADH_2_), as proposed by Fontecave *et al*. 28. Thus, our results suggest that Nox and Npo participate in iron metabolism and oxygen metabolism in the presence of free FAD in *A. xylanus*.

**Table 4:** Ferric reductase activities in the presence or absence of 8 μm free FAD on the cell‐free extract, NADH oxidase, and NAD(P)H oxidoreductase. Data are presented as the mean ± standard deviation from three independent experiments

Electron donor	Iron compounds	Ferric reductase (U·mg^−1^ protein)	
		− FAD	+ 8 μm FAD
NADH	Cell‐free extract		
	Ferric citrate	10.1 × 10^−3^ ± 1.1 × 10^−3^	32.1 × 10^−3^ ± 1.3 × 10^−3^
	NADH oxidase		
	Ferric citrate	0.8 ± 0.2	11.5 ± 2.9
	Fe(III)‐EDTA	0.8 ± 0.1	7.5 ± 0.2
	Fe(III)‐IDA	0.8 ± 0.0	5.4 ± 1.5
	Fe(III)‐NTA	0.7 ± 0.2	10.0 ± 0.4
	Fe(III)‐DTPA	0.4 ± 0.2	12.9 ± 1.7
NADPH	Cell‐free extract		
	Ferric citrate	5.3 × 10^−3^ ± 0.5 × 10^−3^	9.7 × 10^−3^ ± 1.2 × 10^−3^
	NAD(P)H oxidoreductase		
	Ferric citrate	0.1 ± 0.0	0.7 ± 0.1
	Fe(III)‐EDTA	2.6 ± 0.2	3.6 ± 0.1
	Fe(III)‐IDA	0.1 ± 0.0	0.5 ± 0.1
	Fe(III)‐NTA	0.1 ± 0.0	0.6 ± 0.0
	Fe(III)‐DTPA	0.9 ± 0.1	2.2 ± 0.2

In this study, we confirmed that free flavins are present in *A. xylanus*, and FAD is a predominant free flavin species. The physiological concentration of free FAD was sufficient to stimulate molecular oxygen and ferric iron reductase activities of two flavoproteins, Nox and Npo.

**Figure 8:**
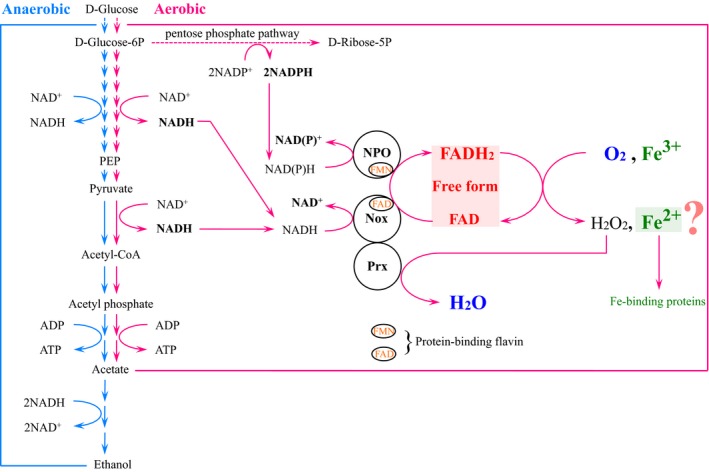
Oxygen and iron metabolisms pathways involve free FAD in *Amphibacillus xylanus*.

*Amphibacillus xylanus* grows well under aerobic conditions using glucose as an energy source, but the energy metabolic system predicted in our previous works lacked an efficient NAD(P)H reoxidation system 17, 40. NAD(P)H should be oxidized to NAD(P)^+^ constantly because NAD(P)^+^ is reused as a substrate in a glycolytic pathway, pyruvate metabolic pathway, and pentose phosphate pathway for the next cycle of metabolism. Based on our results, the free flavin‐associated enzyme system plays a role in the effective NAD(P)H reoxidation system by passing reducing equivalents of NAD(P)H to molecular oxygen or ferric iron via the reduced form of flavin. Thus, in conclusion, this system can also participate in oxygen detoxification and supply ferrous iron during aerobic growth in *A. xylanus* (Fig. 8). However, the simultaneous production of H_2_O_2_ and ferrous iron by free reduced flavin in cells may promote the production of hydroxyl radical via the Fenton reaction, resulting in cell death. Accordingly, both H_2_O_2_ and ferrous iron must be metabolized properly and safely in normal living cells. As described above, H_2_O_2_ is rapidly eliminated by the Nox‐Prx system. Furthermore, we hypothesized that the free iron in the ferrous form must be chelated to prevent it from interacting with H_2_O_2_ and inducing insolubilization inside the alkaliphile *A. xylanus* 43. However, this bacterium lacks general iron chelators including siderophores. Thus, to better understand the aerobic growth of *A. xylanus*, it is necessary to investigate ferrous iron chelators in *A. xylanus* (Fig. 8).

## Author contributions

YN designed the experiment. DM, SK, JS, KK, YK, KT, and SK acquired and analyzed data. DM, SK, AA, and YN wrote the manuscript.

## Supporting information


**Fig. S1.** SDS/PAGE and spectral analysis of NAD(P)H oxidoreductase. (A) NAD(P)H oxidoreductase protein purified from *A. xylanus* was resolved on a 12.5% SDS/PAGE gel and stained with brilliant blue R. (B) The enzyme showed absorption spectra typical of a flavoprotein with a maximum peak at 441 nm.

**Fig. S2.** *Amphibacillus xylanus* requires iron during aerobic growth. *Amphibacillus xylanus* was aerobically cultured in semidefined medium with 1 ppm iron (+ Fe) or without iron (−Fe) at 39.5°C.

**Fig. S3.** Northern blotting of total RNA proved with the peptide deformylase gene (*def*). *Amphibacillus xylanus* was cultured under 0% (AN) and 21% (AE) oxygen. Total RNA was extracted as described previously [41]. The estimated sizes (kb) are indicated on the left. 23S and 16S rRNA stained with ethidium bromide are presented below the autoradiogram.
