# Supplementary figures and images for: Intracellular free flavin and its associated enzymes participate in oxygen and iron metabolism in Amphibacillus xylanus lacking a respiratory chain
Source: FEBS Open Bio. 2018 May 9;8(6):947–61. doi: 10.1002/2211-5463.12425 (PMC5986008; doi:10.1002/2211-5463.12425)

(A)

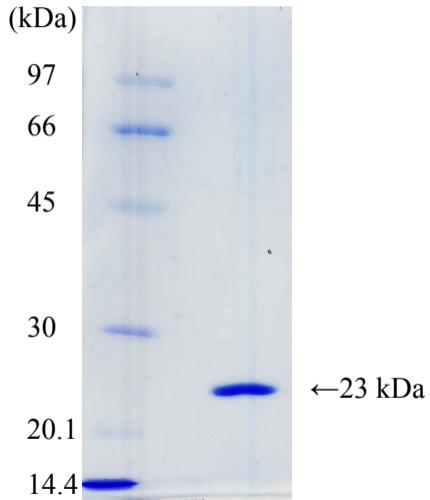

(B)

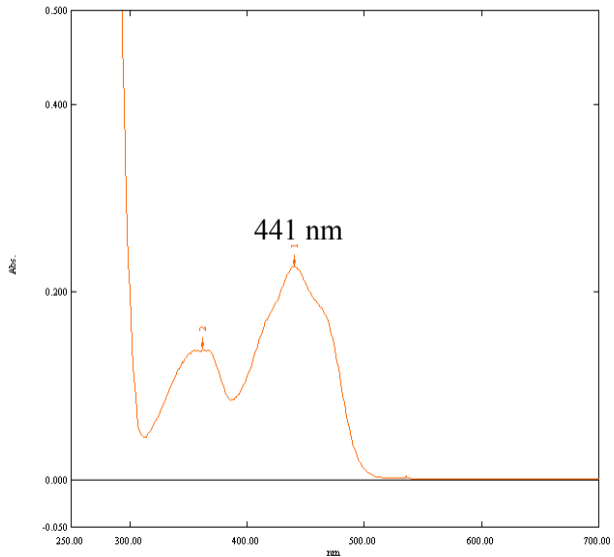

Supplement: Supplementary file 1 — Fig. S1. SDS/PAGE and spectral analysis of NAD(P)H oxidoreductase. (A) NAD(P)H oxidoreductase protein purified from A. xylanus was resolved on a 12.5% SDS/PAGE gel and stained with brilliant blue R. (B) The enzyme showed absorption spectra typical of a flavoprotein with a maximum peak at 441 nm. [file FEB4-8-947-s001.pdf]

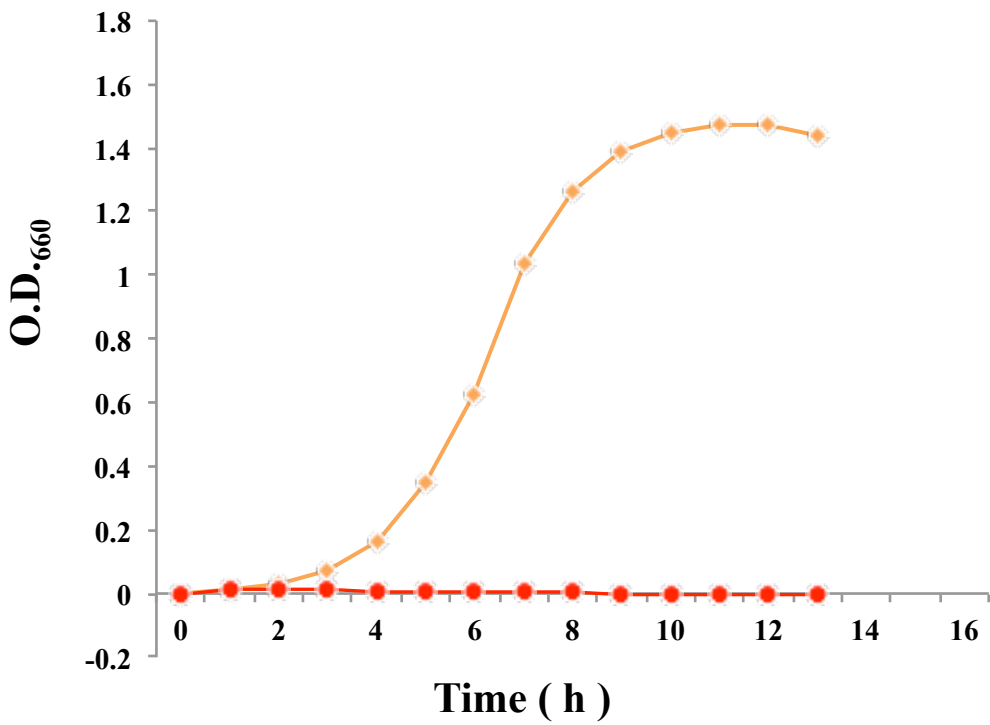

Supplement: Supplementary file 2 — Fig. S2. Amphibacillus xylanus requires iron during aerobic growth. Amphibacillus xylanus was aerobically cultured in semidefined medium with 1 ppm iron (+ Fe) or without iron (−Fe) at 39.5°C. [file FEB4-8-947-s002.pdf]

*def*

AE AN

3.0 kb

2.5 kb

2.0 kb

1.5 kb

rRNA

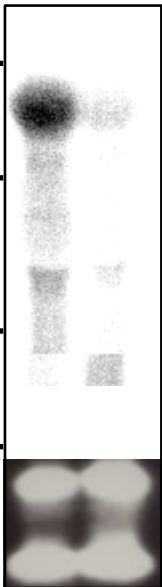

Supplement: Supplementary file 3 — Fig. S3. Northern blotting of total RNA proved with the peptide deformylase gene (def). Amphibacillus xylanus was cultured under 0% (AN) and 21% (AE) oxygen. Total RNA was extracted as described previously [41]. The estimated sizes (kb) are indicated on the left. 23S and 16S rRNA stained with ethidium bromide are presented below the autoradiogram. [file FEB4-8-947-s003.pdf]
